# High expression of BCL-2 predicts favorable outcome in non-small cell lung cancer patients with non squamous histology

**DOI:** 10.1186/1471-2407-10-186

**Published:** 2010-05-09

**Authors:** Valsamo K Anagnostou, Frank J Lowery, Vassiliki Zolota, Vassiliki Tzelepi, Arun Gopinath, Camil Liceaga, Nikolaos Panagopoulos, Konstantina Frangia, Lynn Tanoue, Daniel Boffa, Scott Gettinger, Frank Detterbeck, Robert J Homer, Dimitrios Dougenis, David L Rimm, Konstantinos N Syrigos

**Affiliations:** 1 Department of Pathology, Yale University School of Medicine, New Haven, CT, USA; 2 Department of Pathology, University of Patras, Patras, Greece; 3 Cardiothoracic Surgery Department, University of Patras, Patras, Greece; 4 Department of Pathology, Sotiria General Hospital, Athens, Greece; 5 Section of Pulmonary and Critical Care Medicine, Yale University School of Medicine, New Haven, CT, USA; 6 Department of Surgery, Yale University School of Medicine, New Haven, CT, USA; 7 Section of Medical Oncology, Department of Internal Medicine, Yale Cancer Center, Yale University School of Medicine, New Haven, CT, USA

## Abstract

**Background:**

Bcl-2 promotes cell survival by inhibiting adapters needed for the activation and cleavage of caspases thus blocking the proteolytic cascade that ultimately dismantles the cell. Bcl-2 has been investigated as a prognostic factor in non small cell lung cancer (NSCLC) patients with conflicting results.

**Methods:**

Here, we quantitatively assessed Bcl-2 expression in two large and independent cohorts to investigate the impact of Bcl-2 on survival. AQUA^®^, a fluorescent-based method for analysis of in situ protein expression, was used to measure Bcl-2 protein levels and classify tumors by Bcl-2 expression in a cohort of 180 NSCLC patients. An independent cohort of 354 NSCLC patients was used to validate Bcl-2 classification and evaluate outcome.

**Results:**

Fifty % and 52% of the cases were classified as high expressers in training and validation cohorts respectively. Squamous cell carcinomas were more likely to be high expressers compared to adenocarcinomas (63% vs. 45%, p = 0.002); Bcl-2 was not associated with other clinical or pathological characteristics. Survival analysis showed that patients with high BCL-2 expression had a longer median survival compared to low expressers (22 vs. 17.5 months, log rank p = 0.014) especially in the subset of non-squamous tumors (25 vs. 13.8 months, log rank p = 0.04). Multivariate analysis revealed an independent lower risk for all patients with Bcl-2 expressing tumors (HR = 0.53, 95% CI 0.37-0.75, p = 0.0003) and for patients with non-squamous tumors (HR = 0.5, 95% CI 0.31-0.81, p = 0.005).

**Conclusions:**

Bcl-2 expression defines a subgroup of patients with a favorable outcome and may be useful for prognostic stratification of NSCLC patients.

## Background

Bcl-2 is a key regulator of the mitochondrial apoptotic pathway promoting survival by inhibition of adapters necessary for the activation and cleavage of caspases [1-4]. It is the balance and competitive dimerization between anti-apoptotic (Bcl-2, Bcl-X_L_, Bcl-W, Mcl-1, A1) and pro-apoptotic (Bax, Bak, Bad, Bid) Bcl-2 family members that determines cell fate and regulates the response to apoptotic signals [2,5,6]. Some tumors evade apoptosis and obtain a survival advantage through aberrant Bcl-2 expression and the oncogenic potential of Bcl-2 is well documented in follicular lymphomas [7]. Bcl-2 has been found to induce tumor growth and confer resistance to chemotherapeutic agents in xenograft models of human non small cell lung cancer (NSCLC) [8-10] and Bcl-2 inhibition seems to contribute to reversal of drug resistance in a variety of cancer cell line models[11,12]. Demonstration of the role of Bcl-2 in oncogenesis has prompted the development of drugs targeting Bcl-2 for degradation[13]. Despite the promising preclinical studies supporting a critical role of Bcl-2 in chemoresistance, addition of anti-Bcl-2 antisense oligonucleotide therapies to standard chemotherapy was not correlated with a survival benefit [14-18].

Bcl-2 has been found to be upregulated in a series of tumor types including squamous cell carcinomas of the lung and the potential of Bcl-2 to predict survival for NSCLC patients has been assessed by immunohistochemistry, with different studies demonstrating varying degrees of correlation between Bcl-2 expression and outcome [19-28]. A meta-analysis of 28 clinical studies showed that Bcl-2 positivity was associated with improved survival with a combined hazard of 0.71[22], however numerous individual studies suggest that Bcl-2 has no prognostic value for NSCLC [20,21,27-31] while others report a correlation with poor prognosis [32-36]. These conflicting observations are mainly attributed to small sample size, different immunohistochemical techniques and qualitative scoring systems for Bcl-2 expression, lack of reproducibility and most importantly lack of independent validation of the results. Here we studied the level of Bcl-2 expression in lung cancer patients and assessed its prognostic impact on a large cohort of primary NSCLC; our findings were then validated in an independent cohort of 354 NSCLC patients from another continent.

## Methods

### Cohorts

Formalin-fixed paraffin-embedded primary NSCLC tumors from 180 patients that underwent surgery at Yale University/New Haven Hospital between January 1995 and May 2003 were obtained from the archives of the Pathology Department of Yale University (New Haven, CT). The Yale University cohort consisted of 92 (51%) men and 88 (49%) women with median age of 65 years. Data on stage according to TNM system and differentiation and histological type according to the World Health Organization (WHO) classification for NSCLC[37] is shown in Table 1. All patients were treatment-naïve prior to tumor resection (or acquisition of surgical biopsies for stage IV patients); average follow-up time was 39.32 ± 2.2 months (median 27.3, range 0.1-182). In parallel, we assessed a retrospectively collected, independent cohort of 354 NSCLC patients diagnosed between 1991 and 2001, obtained from the Pathology Departments of Sotiria General Hospital (Athens, Greece) and Patras University General Hospital (Rion, Greece). This cohort consisted of 290 (82%) men and 42 (12%) women with median age of 64 years. Details on stage, differentiation and histological type are shown in Table 1. Three hundred and forty three patients had undergone complete surgical resection for NSCLC and 343 patients received no chemotherapy or radiation prior to resection. Average duration of follow up in this cohort was 24.8 ± 1.1 months (median 20, range 0.1-223). The study was approved by the institutional review boards of all centers. Written informed consent was obtained for each case prior to inclusion in the study.

**Table 1:** Clinical characteristics and BCL-2 expression for the training and validation cohorts.

	N	(%)	BCL-2 (mean ± SE)	p value*	N	(%)	BCL-2 (mean ± SE)	p value*
**All**	180	100			354	100		

								

**Age^†^**	64.3 ± 0.8				62.3 ± 0.5			

								

**Sex**								

Male	92	51	44.3 ± 7.7	0.15	290	82	23.72 ± 2.6	0.16

Female	88	49	68.5 ± 15.2		42	12	32.29 ± 2	

Missing	-				22	6		

								

**Stage**								

I	90	50	57.9 ± 11.6	0.39	107	30	30 ± 3.1	0.8

II	27	15	54.5 ± 21		90	25	30.1 ± 3.2	

III	37	21	54.4 ± 21		95	27	32.5 ± 3.7	

IV	16	9	18.7 ± 1.2		38	11	26.7 ± 5	

Missing	10	5			24	7		

								

**Differentiation**								

High	12	7	27.1 ± 5.3	0.22	23	7	27.6 ± 5.4	0.11

Moderate	44	24	63 ± 18.7		138	39	26.32 ± 2	

Low	70	40	39.9 ± 7.8		168	48	34.13 ± 3	

Missing	54	29			25	6		

								

**Histology**								

Adenocarcinoma	111	62	41.9 ± 8.7	0.49	134	38	24.5 ± 2.2	0.07

Squamous cell carcinoma	37	21	57.9 ± 19.5		161	46	35 ± 2.8	

Large cell carcinoma	11	6	85.5 ± 45		5	1	30.4 ± 11.2	

Adenosquamous carcinoma	13	7	37.8 ± 18.4		4	1	25.1 ± 6.7	

Giant cell carcinoma	-				17	5	27 ± 6.6	

Various	8	4			10	3		

Missing	-				23	6		

Abbreviations: SE; standard error

*p is given for t-test for comparison of continuous BCL-2 AQUA scores between 2 groups and for Analysis of Variance (ANOVA) for comparisons among more than 3 groups

^†^Mean value and standard error of the mean are displayed for age in both cohorts

### Tissue Microarrays (TMAs)

Tissue specimens were prepared in a tissue microarray (TMA) format: representative tumor areas were obtained from formalin fixed paraffin embedded specimens of the primary tumor and two 0.6 mm cores from each tumor block were arrayed in a recipient block. Formalin fixed paraffin embedded cell line pellets were used as controls on a smaller specialized TMA (control array): BT-20, MB175, T47D, HT29, SW-480, H1666, SKBR3, MB453, H2126, SKOV3, HCC2279, A431, MB231, MB435, MB436, SUM159, BT474, MCF7 and ZR-751 were purchased from the American Type Culture Collection (Manassas, VA) or donated by other labs. Culture conditions and cell-line TMA construction have been published in detail elsewhere [38].

### Antibodies and Immunohistochemistry

The arrays were deparaffinized with xylene, rehydrated and antigen-retrieved by pressure cooking for 15 minutes in 10 mM Tris/1 mM EDTA buffer (pH = 9). Slides were pre-incubated with 0.3% bovine serum albumin (BSA) in 0.1 M tris-buffered saline (TBS, pH = 8) for 30 minutes at room temperature. Slides were then incubated with a cocktail of the Bcl-2 primary antibody (mouse monoclonal, clone 124, Dako, Carpinteria, CA) and a wide-spectrum rabbit anti-cow cytokeratin antibody (Z0622, Dako, Carpinteria, CA) both diluted 1:100 in BSA/TBS overnight at 4°C. This was followed by an 1-hour incubation with Alexa 546-conjugated goat anti-rabbit secondary antibody (A11010, Molecular Probes, Eugene, OR) diluted 1:100 in mouse EnVision reagent (K4001, Dako, Carpinteria, CA). Cyanine 5 (Cy5) directly conjugated to tyramide (FP1117, Perkin-Elmer, Boston, MA) at a 1:50 dilution was used as the fluorescent chromagen for target detection. Prolong mounting medium (ProLong Gold, P36931, Molecular Probes, Eugene, OR) containing 4',6-Diamidino-2-phenylindole (DAPI) was used to identify tissue nuclei. Lymphoid tissue was used as positive control as indicated by the manufacturer. Negative control sections, in which the primary antibody was omitted, were used for each immunostaining run.

### Standardization

Serial sections of a smaller specialized TMA (control array) were stained aside both cohorts to confirm assay reproducibility; this allows for the construction of a normalization standard curve to adjust for run to run variability. Nineteen cell line formalin fixed paraffin pellets were used as controls and 30 NSCLC patient cases were also included on the control TMA to supplement the cell line controls, those index cases display much higher cut-to-cut and core-to-core reproducibility.

### AQUA^®^

Automated Quantitative Analysis (AQUA) allows exact measurement of protein concentration within subcellular compartments, as described in detail elsewhere[39]. In brief, a series of high resolution monochromatic images were captured by the PM-2000™ microscope (HistoRx, New Haven, CT). For each histospot, in- and out-of-focus images were obtained using the signal from the DAPI, cytokeratin-Alexa 546 and Bcl-2-Cy5 channel. Bcl-2 was measured using a channel with emission maxima above 620 nm, in order to minimize tissue autofluorescence. Tumor was distinguished from stromal and non-stromal elements by creating an epithelial tumor "mask" from the cytokeratin signal. This created a binary mask (each pixel being either "on" or "off") on the basis of an intensity threshold set by visual inspection of histospots. AQUA score of Bcl-2 in each subcellular compartment was calculated by dividing the Bcl-2 compartment pixel intensities by the area of the compartment within they were measured. AQUA scores were normalized to the exposure time and bit depth at which the images were captured, allowing scores collected at different exposure times to be directly comparable.

### Statistical analysis

Pearson's correlation coefficient (R) was used to assess the correlation between AQUA scores from redundant tumor cores as well as the same cores on serial cuts of the control array. An R^2 ^greater than 0.4 was indicative of good inter- and intra-array reproducibility and thus the average values for Bcl-2 AQUA scores from duplicate samples were calculated and treated as independent continuous variables. Analysis of variance (ANOVA) was used to compare continuous AQUA scores among groups with different clinical/pathologic characteristics and chi square was employed for comparison of binarized Bcl-2 AQUA scores between adenocarcinomas and squamous cell carcinomas. X-tile software[40] was used to select the optimal Bcl-2 concentration cutpoint for the Yale University cohort (training set); this cutpoint was subsequently validated in the Greek Lung Cancer cohort (validation set). Survival curves for the validation set were constructed using the Kaplan-Meier method and survival differences were analyzed by the log rank test. Cox proportional hazards regression analysis was used to determine which independent factors jointly had a significant impact on overall survival. All *p *values were based on two-sided testing and differences were considered significant at *p *< 0.05. All statistical analyses were done using the SPSS software program (version 13.0 for Windows, SPSS Inc., Chicago, IL).

## Results

### Assay reproducibility and assessment of Bcl-2 in NSCLC and cell line controls

**Figure 1:**
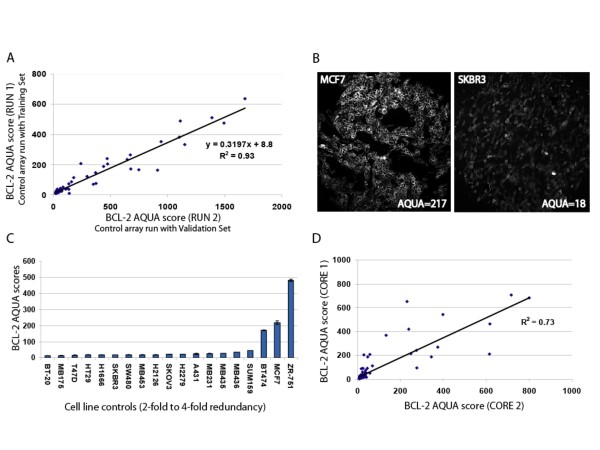
**Distribution and reproducibility of Bcl-2 AQUA scores in cell lines and training set**. A. Plotting AQUA scores of the same histospots on serial cuts of the control array stained aside the 2 cohorts at different runs provided a standard curve in order to normalize for run to run variability (Pearson's R = o.96 between runs, p < 0.0001) B. Distribution of Ncl-2 AQUA scores in 19 cell line controls embedded in the control TMA C. Representative immunofluorescence staining in a high (MCF7) and low (SKBR3) Bcl-2 expressing breast cancer cell lines; AQUA scores are displayed in insets D. Linear regression between Bcl-2 AQUA scores of redundant tumor cores reveals significant correlation (Pearson's R = 0.85, p < 0.0001).

**Figure 2:**
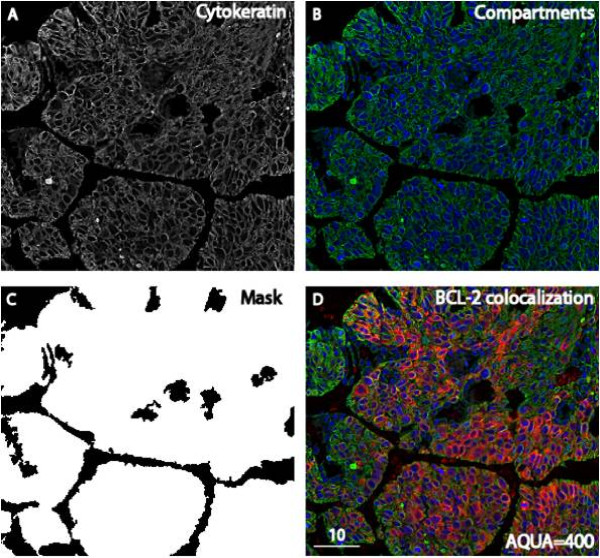
**Automated Quantitative Analysis (AQUA) of Bcl-2 in lung cancer**. (A-D) Tumor histospot corresponding to a NSCLC patient with Bcl-2 AQUA score of 400 A. Cytokeratin-Cy3 image used to identify tumor; the epithelial tumor "mask" is displayed in C B. Colocalization of subcellular compartments: the non nuclear (green) and nuclear (blue) compartment are defined by the cytokeratin and the DAPI signal respectively D. Bcl-2-Cy5 (red), cytokeratin (green) and DAPI (blue) colocalization; Bcl-2 shows a representative cytoplasmic pattern; AQUA score is inset into the bottom right corner.

Evaluation of the inter-array reproducibility for antibody validation did not reveal significant differences between serial sections of the control array (Pearson's R = 0.96, p < 0.0001; Figure 1A). Bcl-2 AQUA scores for specimens on the validation cohort were converted according to the cell line standard curve (y = 0.3197x+8.8; Figure 1A), in order to normalize for run to run variability. Bcl-2 was predominantly expressed in the cytoplasm of cell lines (Figure 1B-C) as well as NSCLC tumor cells (Figure 2) and this cytoplasmic pattern is consistent with its localization in the inner mitochondrial membrane [41]. SKBR3, a known low Bcl-2 expressing breast cancer cell line Bcl-2[42] had an AQUA score of 18, whereas MCF7 and BT474 that are high Bcl-2 expressers [42] showed the highest AQUA scores for Bcl-2 expression (Figure 1B-C).

To assess intra-tumor heterogeneity for Bcl-2 expression, we compared AQUA scores from redundant tumor cores and observed significant correlation (Pearson's R = 0.85, p < 0.0001; Figure 1D and Pearson's R = 0.94, p < 0.0001 for the training and validation cohort respectively). Therefore, AQUA scores in the tumor "mask" were averaged between redundant histospots and final scores ranged from 7.7 to 800 (mean 56 ± 8.4, median 18.8) and from 11.6 to 227 (mean 30 ± 1.7, median 19) for the training and validation set. Specimens with less that 5% tumor area per spot were not included in automated quantitative analysis for not being representative of the corresponding tumor specimen.

### Optimal cut point selection for BCL-2 expression

We applied X-tile[40] in order to determine the optimal cutpoint of continuous Bcl-2 AQUA scores; this statistical method assesses every division of continuous variables into ordinal classes and performs standard Monte Carlo simulations to produce chi squared values which can be maximized to find the optimal cut point in continuous data. Since it is not statistically valid to test multiple divisions with corrections for multiple sampling, rigorous statistical evaluation is achieved by defining divisions in a training set and then validating them in an independent non-overlapping validation set. On that basis we used the Yale University Cohort as a training set to generate a cutpoint that provides the optimal separation in terms of survival - an AQUA score of 18.8 was thus selected- and validated this cutpoint on the Greek Lung Cancer Cohort (validation set).

### Patient characteristics and correlation with Bcl-2 expression

Clinical and pathologic parameters that were analyzed included age, gender, histological type, tumor differentiation and stage. In the training cohort, Bcl-2 expression was identified in 90 (50%) of NSCLCs, including 51 (46%) adenocarcinomas (AC), 23 (62%) squamous cell carcinomas (SCC), 5 (45%) large cell carcinomas (LCC) and 6 (46%) adenosquamous carcinomas (AS). No significant difference in continuous Bcl-2 scores was found among different histological subgroups (mean AQUA scores ± standard error-SE 41.9 ± 8.7, 57.9 ± 19.5, 85.5 ± 45 and 37.8 ± 18.4 for AC, SCC, LCC and AS respectively, ANOVA p = 0.49; Table 1); however when scores were binarized by the optimal cutpoint (AQUA score 18.8) we observed higher Bcl-2 expression in SCC compared to AC (62% vs. 46%, chi square p = 0.08). Bcl-2 was not significantly associated with any other clinicopathological characteristic (Table 1).

In the validation cohort, high BCL-2 expression was observed in 186 (52%) patients: 60 (45%) AC, 101 (63%) SCC, 2 (40%) LCC and 2 (50%) AS were classified as high expressers. Comparison of continuous BCL-2 scores among different histological subgroups revealed higher BCL-2 expression in the SCC group (mean AQUA scores ± SE, 24.5 ± 2.2, 35 ± 2.8, 30.4 ± 11.2, 25.1 ± 6.7 and 27 ± 6.6 for AC, SCC, LCC and AS respectively, ANOVA p = 0.07; Table 1); this was more evident for binarized BCL-2 AQUA scores (63% BCL-2 positive SCC vs. 45% BCL-2 positive AC, chi square p = 0.002). No significant correlation was found between BCL-2 expression and gender, age, stage or tumor differentiation (Table 1).

### Correlation of Bcl-2 with survival (training cohort)

**Figure 3:**
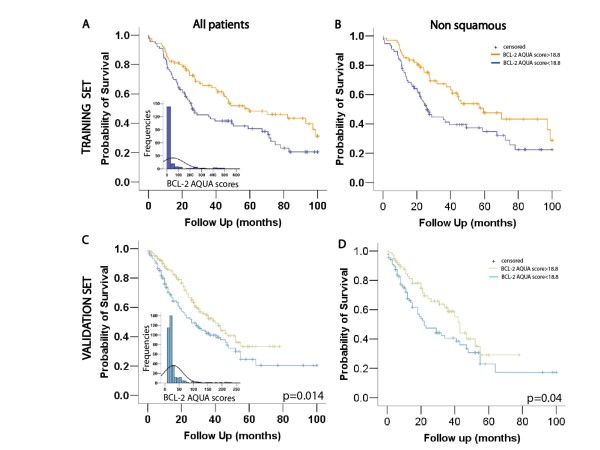
**Disease outcome by Bcl-2 expression in NSCLC**. A, B. Survival curves based on cohort division by the optimal cutpoint generated from the Yale University Cohort (training set); NSCLC and non squamous patients classified as high Bcl-2 expressers (n = 90 and n = 67 respectively) show a benefit towards survival. The distribution of AQUA scores is displayed in inset. C. Kaplan-Meier graphical analysis of survival in NSCLC of the Greek Lung Cancer Cohort (validation set, n = 354). Patients with a high Bcl-2 score (n = 186) had a significant higher median survival compared to the low Bcl-2 group (log rank p = 0.014). The distribution of Bcl-2 AQUA scores is displayed in inset. D. Survival curves for non squamous tumors of the validation cohort classified as high (n = 76) versus low Bcl-2 expressers showed a significant benefit towards survival for the high expressing group (log rank p = 0.04). AQUA; automated quantitative analysis.

NSCLC patients classified as high Bcl-2 expressers (n = 90) had a better prognosis compared to the low expressing group (median survival 38.7 vs. 24.37 months; Figure 3A). Cox univariate analysis with binarized Bcl-2 scores revealed that high Bcl-2 expression resulted in 41% reduction in risk for all NSCLC patients (hazards ratio-HR = 0.59, 95% confidence interval-CI 0.4-0.87, p = 0.009). A subgroup analysis of non squamous tumors yielded similar results; high Bcl-2 expressers (n = 67) showed a longer survival (median survival 31.92 vs. 23.62 months for the high and low group respectively; Figure 3B) and a 44% reduction in risk for death (HR = 0.56, 95% CI 0.35-0.89, p = 0.014). The small number of SCC patients did not allow us to perform meaningful survival analysis for that group.

### Validation of cutpoint selection (validation cohort)

The cutpoint generated from the training set was applied in the validation cohort and we subsequently assessed the potential of Bcl-2 to predict survival; among all patients tumors with high Bcl-2 expression (n = 186) had a higher median survival compared to the low Bcl-2 expressers (median survival 22 vs. 17.5 months respectively, log rank p = 0.014; Figure 3C). Cox univariate analysis with continuous Bcl-2 scores binarized by the optimal cutpoint showed that high Bcl-2 expression resulted in 33% reduction in risk (HR = 0.67, 95% CI 0.49-0. 29, p = 0.016) for all patients of the validation cohort. The same trend was observed in a subgroup analysis for non squamous tumors: high Bcl-2 expressers (n = 76) had a significantly better outcome compared to the low Bcl-2 group (median survival 25 vs. 13.8 months, log rank p = 0.04; Figure 3D) and Bcl-2 expression was associated with a 37% reduction in risk (HR = 0.63, 95% CI 0.4-0.98, p = 0.04). Interestingly no survival benefit was found for the high expressing Bcl-2 squamous cell carcinomas (median survival 26 vs. 21 months for the high and low expressers respectively; log rank p = 0.3; data not shown).

### Assessment of the independent potential of Bcl-2 to predict survival

Multivariate Cox proportional hazards regression analysis was performed to derive risk estimates related to survival for age, gender, stage, tumor differentiation, histological type and Bcl-2 expression; cases with missing values were excluded from analysis (Table 2). Age, stage and Bcl-2 expression were significantly associated with survival and Bcl-2 expression retained its prognostic value such that NSCLC patients with high Bcl-2 expression had a 47% reduction in risk (HR = 0.53, 95% CI 0.37-0.75, p = 0.0003; Table 2). In a subgroup analysis of non squamous tumors, high Bcl-2 expression significantly contributed to the prognostic value of the model revealing the independent favorable prognostic value of Bcl-2 (HR = 0.5, 95% CI 0.31-0.81, p = 0.005; Table 2). Gender, stage, histological type and tumor differentiation were analyzed as categorical and age as continuous variables.

**Table 2:** Multivariate analysis of overall survival for Bcl-2 expression in lung cancer patients of the validation cohort

	Validation Cohort			
	**All patients**		**Non squamous**	

**Characteristic**	**HR (95% CI)**	p value*	**HR (95% CI)**	p value*

				

**Age**	1.02 (1-1.04)	**0.02**	1.03 (1-1.06)	**0.04**

				

**Gender**				

Female	1			

Male	0.72 (0.44-1.18)	0.19	0.6 (0.29-1.24)	0.17

				

**Stage**				

Stage I	1		1	

Stage II	1.9 (1.18-3)	**0.008**	2.2 (1.07-4.54)	**0.03**

Stage III	3 (1.9-4.6)	**< 0.001**	3.6 (1.8-7.1)	**< 0.001**

Stage IV	4.1 (2.4-7.02)	**< 0.001**	5.1 (2.5-10.49)	**< 0.001**

				

**Histology**				

Adenocarcinoma	1			

Squamous cell carcinoma	1.1 (0.76-1.6)	0.6		

Large cell carcinoma	0.8 (0.19-3.46)	0.8		

Adenosquamous carcinoma	0.9 (0.21-3.77)	0.9		

Giant cell carcinoma	1.05 (0.46-2.41)	0.9		

Various	1.5 (0.6-3.97)	0.4		

				

**Differentiation**				

High	1		1	

Moderate	2.1 (0.99-4.6)	0.05	3.01 (1.14-7.89)	**0.025**

Low	1.4 (0.67-3.03)	0.3	1.38 (0.52-3.67)	0.5

				

**Bcl-2**				

Low	1		1	

High	0.53 (0.37-0.75)	**0.0003**	0.5 (0.31-0.81)	**0.005**

*p is given for Cox multivariate analysis, statistically significant p values (p < 0.05) are in boldface, trending p values in italics, HR; hazard ratio

## Discussion

Despite the large number of studies the value of Bcl-2 for predicting outcome for NSCLC remains controversial. Meta-analysis of 13 immunohistochemical studies assessing Bcl-2 with the same antibody clone as used in this study revealed that Bcl-2 was associated with better survival for NSCLC patients (HR = 0.71, 95% CI 0.61-0.83) however this study was biased in favor of positive trials [22]. Bcl-2 has also been reported to be a negative prognostic marker [32,33,35,36,43] whereas the majority of studies on Bcl-2 expression show no impact on survival [20,21,27,28]. Methodology differences in immunohistochemistry, different thresholds for Bcl-2 and use of conventional semi-qualitative, subjective pathologist-scoring systems, lack of reproducibility and lack of independent validation of the results preclude firm conclusions based on the current literature. The goal of this work was to quantitatively and objectively assess Bcl-2 expression in NSCLC to determine its prognostic value.

Bcl-2 expression has been confirmed in 20% to 58% of NSCLC and 32% to 69.4% of squamous cell carcinomas of the lung [19,22,28] and this is consistent with our findings of 50% and 51% Bcl-2 positive NSCLC tumors and 62% and 63% positive squamous cell carcinomas for the training and validation cohorts respectively. Bcl-2 has been found to mediate the anti-apoptotic effect of nicotine in lung cancer [44] and we observed higher Bcl-2 expression in tumors of smokers (n = 265) compared to non smokers (n = 29; mean ± SE 30.8 ± 2.1 and 22.7 ± 2.96 respectively; data not shown) however this did not reach statistical significance due to small number of non smokers.

Given the known anti-apoptotic role of the Bcl-2 one would expect that high Bcl-2 expression would confer to a worse prognosis, rather than a prolonged survival. One possible explanation is that Bcl-2 has a cell cycle inhibitory effect genetically separable from its survival function that could potentially reduce its oncogenic impact [45]. It is possible that the effect of Bcl-2 on apoptosis is countered by other oncogenes (like p53); apoptosis in lung cancer could thus occur independently without being primarily modulated by Bcl-2. Both proapoptotic and antiapoptotic Bcl-2 family proteins are known to change their respective phenotypes in certain scenarios and Bcl-2 appears to have normal cellular functions that promote cell survival [46]. Although Bcl-2 is typically considered to inhibit caspase activation, it can also serve as a downstream "death substrate" converted by caspases to a Bax-like effector and activation of its BH3 homology domain not only inactivates the antiapoptotic activity of Bcl-2 but also releases a potent proapoptotic fragment [47]. In data not shown, we have found a correlation between Bcl-2 and cleaved caspase 3 expression (Spearman rho = 0.147, p = 0.049) and this is consistent with the existence of a feedback loop between Bcl-2 and caspases, allowing the latter to bypass Bcl-2 inhibition. Thus, Bcl-2 may play opposing roles in apoptosis regulation depending on cellular context.

Accurate measurement of Bcl-2 in NSCLC has been historically difficult due to subjective and qualitative methodology complicated by lack of standardization between studies. Here, we employed AQUA[39], a fully quantitative method to calculate Bcl-2 concentration within subcellular compartments and construct a cell line-based standard curve in order to normalize for run to run variability. Furthermore, the controversial impact of Bcl-2 on survival can be partially attributed to the different and often un-justified cut-off points used to classify NSCLC tumors for Bcl-2 positivity. In order to overcome bias related to any single *a priori *cutpoint selection we used X-tile[40] to assess every division of continuous Bcl-2 scores into ordinal classes and generate a cutpoint that provides the optimal classification in terms of survival; interestingly the cut point selected was the median Bcl-2 AQUA score of the training set. However, since we had no a priori biological reason to select the median as the cut-point, we used the training set cohort to prevent operator bias in cut-point selection. This rigorous statistical approach demands independent validation of the results; therefore we validated the prognostic classification generated in an independent NSCLC cohort from a separate institution.

A potential limitation of this study is the retrospective nature of the collection of both cohorts; a prognostic trial of a single marker such as this can rarely be prospectively designed outside the setting of a clinical trial. Furthermore, the 2 cohorts are not well matched in terms of gender and disease stage however we believe that the similarity of the results in both cohorts suggests robustness of the observation.

Squamous cell carcinomas seem to have a distinct molecular background compared to non squamous tumors [48] and this is also prominent in the clinical setting, where squamous histology is an exclusion criterion for bevacizumab [49,50] as well as pemetrexed treatment [51]. We demonstrated a survival benefit for Bcl-2 expression in patients with non squamous histology absent in squamous cell carcinomas and this observation confirms the effect of histology for prognostic stratification of NSCLC patients. Although we are optimistic that the prognostic findings presented here are likely to portend predictive results, it is not evident that Bcl-2 expression as a positive prognostic factor is related to a beneficial therapeutic target potential [14-17]. The inconsistency between pre-clinical studies and clinical trials on Bcl-2 inhibitors confirms the necessity of identifying biomarkers capable of predicting which cancers would benefit from treatment with Bcl-2 inhibitors [18]; whether Bcl-2 expression itself has such a predictive value in non squamous NSCLC remains to be determined.

## Conclusions

The present study is the first one assessing Bcl-2 expression by a fully quantitative method and demonstrating the impact of Bcl-2 on survival of NSCLC in 2 independent cohorts. Our results indicate that loss of Bcl-2 expression is correlated with a more aggressive behaviour of NSCLC tumors and high Bcl-2 expression defines a subgroup of patients with a favourable outcome. Our validated model may be useful for prognostic stratification of lung cancer patients as well as incorporation of Bcl-2 into clinical decisions.

## Competing interests

The authors declare that they have no competing interests.

## Authors' contributions

VA, FJ and CL carried out the immunoassays, VA performed the statistical analysis, VZ, VT, AG and KF participated in the tissue microarray construction for the Greek lung cancer cohort, NP and DD provided the clinical data for the Greek lung cancer cohort, VA, DR and KS drafted the manuscript, VA, LT, DB, SG, FD, RH, DR and KS conceived of the study and participated in the design of the study. All authors read and approved the final manuscript.

## Pre-publication history

The pre-publication history for this paper can be accessed here:

http://www.biomedcentral.com/1471-2407/10/186/prepub
